# Endoscopic Primary Breast Augmentation With Loco-Regional Anesthesia: Preliminary Experience of 200 Consecutive Patients

**DOI:** 10.1093/asjof/ojae033

**Published:** 2024-04-30

**Authors:** Araco Antonino

## Abstract

**Background:**

Breast augmentation with implants recorded over 1.6 billion procedures globally in 2022. To reduce surgical trauma and complications and facilitate a fast recovery, we employ an ultrasound-guided local–regional anesthesia technique, the creation of a partial submuscular implant pocket by direct endoscopic visualization and minimal skin access on the mammary fold.

**Objectives:**

The aim in this study is to evaluate whether breast augmentation performed in endoscopy under local–regional anesthesia reduces postoperative recovery time, reduces complications, and increases patient satisfaction.

**Methods:**

Patients provided their consent through a signed form. We set strict inclusion and exclusion criteria. We prospectively evaluated postoperative pain and recovery times, the rate of complications, and patient satisfaction at 12 months postsurgery.

**Results:**

Between January 2021 and September 2022, 200 patients met the inclusion criteria. The average operation time was 54.2 min. Patients were discharged from the hospital within 2 to 3 h. Eighty-nine percent of patients expressed great satisfaction with the result. None of the patients experienced postsurgical complications.

**Conclusions:**

In our initial study, we showed that endoscopic breast augmentation conducted under localized anesthesia is safe. It allows for quick recovery postsurgery and swift resumption of everyday activities. The overall complication risk is less than what has been reported in scientific studies for the classic dual-plane technique. Moreover, this approach yields excellent patient satisfaction. Additional prospective and randomized studies will be required to enhance the scientific validity of this technique. Moreover, a larger patient cohort will be essential to stratify the risks associated with varying prosthetic volumes.

**Level of Evidence: 4:**

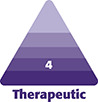

Breast augmentation with implants, being the most common plastic surgery, recorded over 1.6 billion procedures globally in 2022.^1^ Its safety and low complication rate are well documented in scientific literature.^2-4^

Regardless, complications associated with general anesthesia can be life-threatening.^5-8^ We categorize the complications related to breast augmentation as early complications occurring within a week, which include conditions such as hematoma,^9,10^ prosthesis dislocation,^7^ and mammary fold rupture;^11^ often, these result from preparing the pocket for the implant. We also observe medium-term complications within a month, such as seroma,^12,13^ infections,^14-16^ and wound issues with or without implant exposure.^17^ Late complications experienced within a year include late seroma^18,19^ and capsular contracture.^20-22^ Speedy recovery and a swift return to daily routines after surgery have always been vital to patients, and today, these play a crucial role in the decision of patients to undergo surgery.^23^

To reduce surgical trauma and complications and facilitate a swift recovery, we employ a local–regional anesthesia technique. This involves an ultrasound-guided selective blockade of the pectoral nerves and the creation of a partial submuscular implant pocket. This is achieved through direct endoscopic visualization and minimal skin access to the mammary fold. The aim in this study is to evaluate whether breast augmentation performed through the endoscopy technique under local–regional anesthesia reduces postoperative recovery time, reduces complications, and increases patient satisfaction.

## METHODS

### Patient Selection

The study adhered to ethical standards from the 1975 Declaration of Helsinki, aligned with Good Clinical Practice principles.

Two weeks prior to the surgery, patients provided their consent through a signed form. To ensure consistency in terms of positioning and lighting, digital macrophotographs were captured using a D7100 Nikon camera with 12.0 megapixels (Nikon, Tokyo, Japan). For a more comprehensive view, 3-dimensional images were obtained by applying the Vectra H2 camera system (Canfield, Parsippany, NJ), which employs computer-vision technology.

We set strict inclusion criteria: females younger than the age of 40 with a BMI between 19 and 23 who, for the first time, underwent breast augmentation and were hoping for a breast implant smaller than 295 cc. We eliminated the following categories of females from the study: those over the age of 40, those with a BMI lower than 19 or higher than 23, those who have been pregnant at least once, those who have already undergone breast augmentation surgery, and those hoping for a breast implant larger than 295 cc. Furthermore, we ruled out females who needed to combine mastopexy with their implant surgery. We also excluded patients when the distance between the mammary fold and the inferior areola exceeded 6 cm, tuberous breasts, asymmetric breasts requiring different-size implants, those with a narrow or constricted lower breast pole, or those who especially needed access from the areola.

Between January 2021 and September 2022, 382 patients underwent aesthetic breast surgery at our private plastic surgery hospital. Out of these, 200 patients met the inclusion criteria and underwent surgery using local anesthesia and endoscopic techniques.

### Surgical Technique

#### The Block Anesthesia Nerve Pectoralis

**Figure 1.**
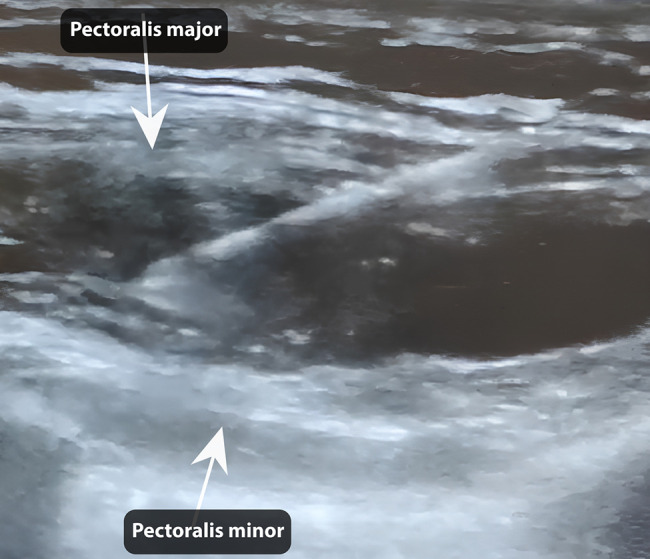
An ultrasound image showing the pectoralis major and minor muscles divided by anesthetic.

In this technique, the patient should be positioned either supine or semi-lateral, with the arm abducted at 90°. The external landmarks to take note of include the clavicle, pectoralis major muscle, and anterior and midaxillary lines. A needle cannula, 25 G in size and 60 mm long, was introduced 3 cm below the clavicle's margin, medial to the thoracoacromial artery, guided by an ultrasound machine with a 5 to 17 MHz multifrequency probe and color Doppler. Afterward, the second and third ribs, along with the major and minor pectoralis muscles, were identified. The area between these 2 muscles was then injected with 40 mg of plain ropivacaine (1 vial of 20 mL) for each side (Figure 1, Video). We avoided the use of epinephrine because of the risk of intramuscular infiltration. Ten milligrams of Midazolam hydrochloride were administered to all patients 10 to 15 min before the commencement of the procedures. A surgical incision was made 15 to 20 min after the ropivacaine injection was administered in order to give enough time for the medication to be effective.

#### Endoscopic Breast Implant Pocket Preparation

**Figure 2.**
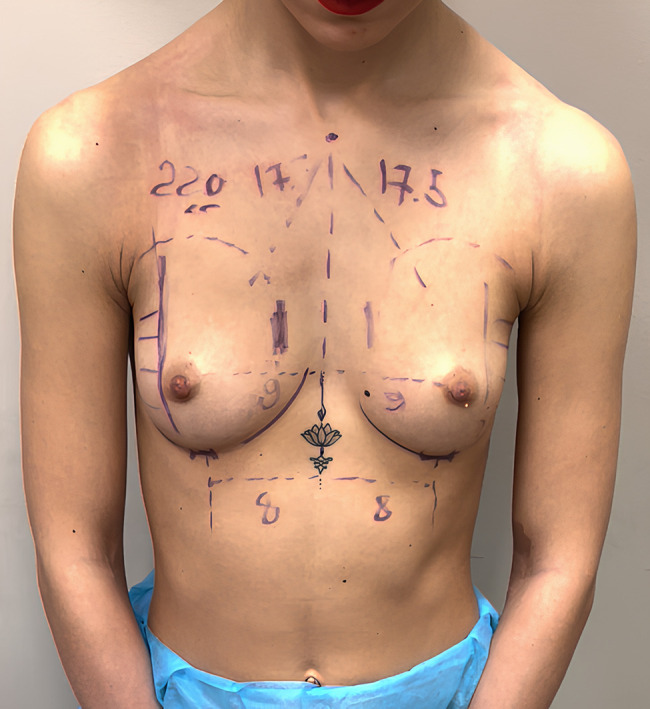
Preoperative markings on a 23-year-old female patient.

**Figure 3.**
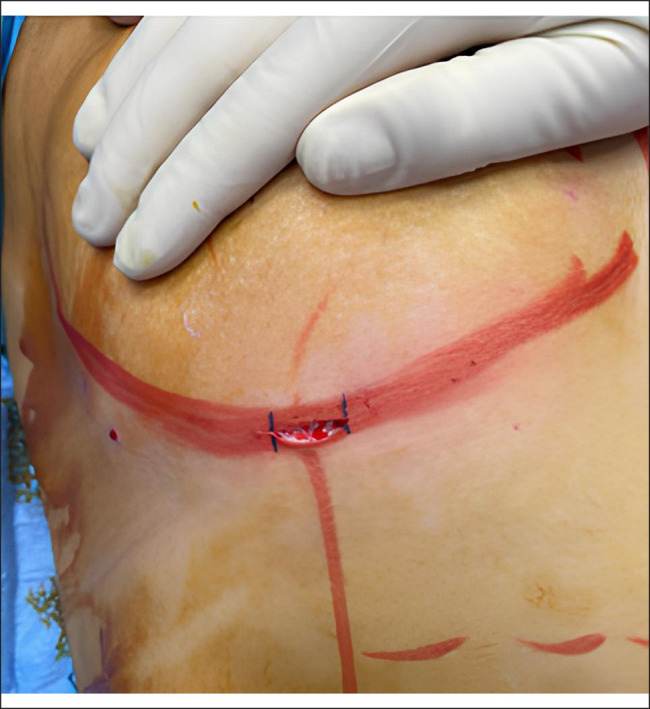
Minimal skin incision shown on a 23-year-old female patient.

Patients were given 2 g of ceftriaxone IV a few minutes before the incision. Those allergic to cephalosporins received 500 mg of ciprofloxacin. All patients underwent a personalized alteration of the dual-plane breast augmentation technique. According to Tebbetts and Adams, the technique involves an incision in the mammary fold and placement of the prosthesis partially underneath the pectoralis major muscle. In our patients, preoperative diagrams were drawn carefully to indicate prosthetic pocket extensions; these did not extend to more than 2 to 3 cm laterally beyond the areola. A skin incision was made exactly on the mammary fold, at an approximate distance of 5 to 6 cm from the lower edge of the areola (Figure 2). After infiltrating the skin with 5 mL of lidocaine (50 mg) for each side, a small skin incision of approximately 2.2 to 2.7 cm (average 2.4 cm) was made (Figure 3). The lower edge of the pectoralis major muscle was identified; it was opened, the endoscope was inserted, and the pocket preparation was continued superiorly under direct vision.

**Figure 4.**
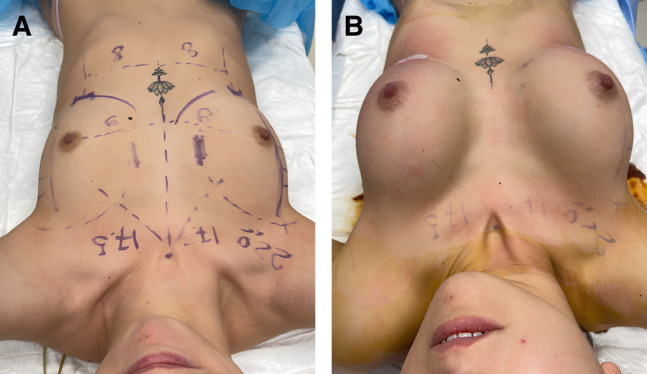
A 23-year-old female patient shown (A) before implant insertion and (B) after implant insertion.

**Figure 5.**
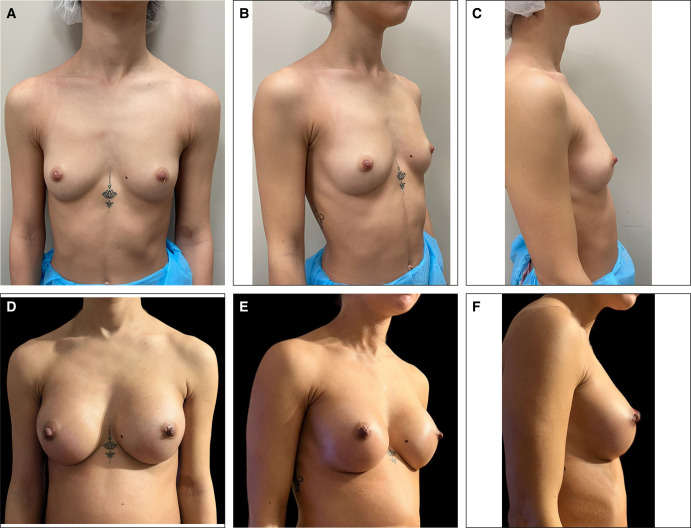
Patient 1 (a 23-year-old female): (A) before operation, frontal view; (B) before operation, oblique view; (C) before operation, lateral view; (D) 12 months after operation, frontal view; (E) 12 months after operation, oblique view; and (F) 12 months after operation, lateral view.

The pectoralis major muscle was gently detached from the thoracic fascia using a Tebbetts hand-activated monopolar insulated forceps. In the lower medial part of the breast pole, the pectoral muscle was released from the ribs, while ensuring that a thin layer of muscle fibers covered the prosthesis to prevent median rippling. Laterally, a segment of the pectoralis major muscle was preserved to stabilize the prosthesis and prevent lateral displacement. The size of the pocket created was smaller than that of the breast implant, because the latter would eventually conform to the final pocket size (Figures 4, 5). After the implant pocket was created, it was rinsed with 50 mL of saline solution. Patients were fitted with smooth round silicone implants (Motiva Ergonomix—Establishment Labs, Costa Rica) ranging in size from 220 to 295 cc (average 245 cc). These implants were inserted using a Keller Funnel device (Abbvie, North Chicago, IL).

We did not insert a drain into or apply a compression bandage on any of the patients. Postoperative follow-ups are scheduled after 7, 14, and 30 days and then at 3, 6, and 12 months.

#### Assessment of Efficacy and Tolerability

Our first objective was to prospectively evaluate postoperative pain at 12, 24, 48, and 72 h intervals using a postoperative pain scale (Table 1). We also aimed to determine recovery times at 24 and 48 h, as well as 7, 14, and 30 days postsurgery using the postoperative recovery scale (Table 2).

**Table 1.** Postoperative Pain Scale

	Score
I have no pain and I don’t need to take painkillers	0
I have minimal pain, I can bear it and I don’t need painkillers	2
I have minimal pain that subsides with 400 mg of ibuprofen	4
I have moderate pain that subsides with 800 mg of ibuprofen	6
I have severe pain that subsides with 1.6 g of ibuprofen (800 mg every 12 h)	8
I have severe pain that subsides with 1.6 g of ibuprofen (800 mg every 12 h) plus 1 g of paracetamol (500 mg every 12 h)	10

**Table 2.** Postoperative Recovery Scale

	Score
I can’t make any effort; I can’t get dressed because the movement of my arms causes me pain	0
I can move my arms with difficulty—I am independent only in the house	2
I can drive the car	4
I resume my work activities	6
I resume sporting activities without straining my arms and pectoral muscles	8
I resume sporting activities with full use of my arms and pectoral muscles	10

The second objective was to gauge the rate of complications 7 days (early complications), 30 days (intermediate complications), and 12 months (late complications) after surgery.

Finally, the third objective focused on measuring patient satisfaction 12 months’ postsurgery, using the patient satisfaction questionnaire (Table 3). The 3 rating scales used were designed by me.

**Table 3.** Patient Satisfaction Questionnaire

	Score
I look worse than before; my breasts are asymmetric/of strange shape	0
I do not notice any acceptable difference; the breasts look very similar to those before in terms of shape/size	2
The cosmetic result is under my expectation; I have not achieved the desired breast shape/size	4
The cosmetic result is acceptable, but I would have expected more in terms of breast shape/size	6
The cosmetic result is according to my expectation in terms of shape and size. I would recommend this surgery to my relatives	8
The cosmetic result is beyond my expectation in terms of shape and size. At least one of my friend/family members underwent the same operation after my recommendation	10

## RESULTS

The average age of the patients was 24.3 years (range, 18-34 years); the average operation time was 54.2 min (range, 42-70 min). The patients were discharged from the hospital within 2 to 3 h (an average of 2.4 h) and prescribed 1 g of amoxicillin + clavulanic acid a day (500 mg of ciprofloxacin a day if allergic to cephalosporins) and an ibuprofen 200 mg capsule for 6 days. Table 4 summarizes the demographics and clinical characteristics of these patients. On average, the follow-up period was 17.4 months, ranging from 14 to 24 months.

**Table 4.** Patient Data

*n*	Age	BMI	Implant size (cc)
200	28.43 ± 5.34 (18-40)	22.87 ± 0.43 (19.3-22.7)	265 ± 22 (220-295)

### Primary End Point

After surgery, 13 patients (6.5%) needed 800 mg of ibuprofen at the 12 h mark. At the 24 h mark, 3 patients (1.5%) needed 800 mg and 14 patients (7%) needed 400 mg. By the 48 h mark, only 1 patient (0.5%) needed 800 mg, 3 patients (1.5%) needed 400 mg, and 26 patients (13%) needed 200 mg. By 72 h, none of the patients required painkillers. Only 6.5% of patients experienced the highest level of postoperative pain, a score of 6, within 12 h of the operation. After 48 h, only 1 patient (0.5%) still reported this level of pain (Table 5). All patients were discharged from the hospital 3 h postsurgery.

**Table 5.** Results: Postoperative Pain

	Score	12 h post	24 h post	48 h post	72 h post
Mild	0	0	90 (45%)	170 (85%)	200 (100%)
	2	152 (76%)	93 (46.5%)	26 (13%)	0
Moderate	4	35 (17.5%)	14 (7%)	3 (1.5%)	0
	6	13 (6.5%)	3 (1.5%)	1 (0.5%)	0
Severe	8	0	0	0	0
	10	0	0	0	0

Seventy-one percent of patients were already driving a car, and 6% were returning to work within 24 h. All patients resumed a working life within 48 h, only avoiding excessive effort. Sixty-three percent resumed regular sporting activity with the use of their arms and chest, but avoiding excessive power and strong muscle use within 15 days, and all patients resumed full sports activity within 30 days (Table 6).

**Table 6.** Results: Postoperative Recovery

Score	24 h post	48 h post	7 days	15 days	30 days
0	0	0	0	0	0
2	6 (3%)	0	0	0	0
4	142 (71%)	0	0	0	0
6	12 (6%)	200 (100%)	200 (100%)	0	0
8	0	0	0	126 (63%)	7 (3.5%)
10	0	0	0	0	193 (96.5%)

### Secondary End Points

**Figure 6.**
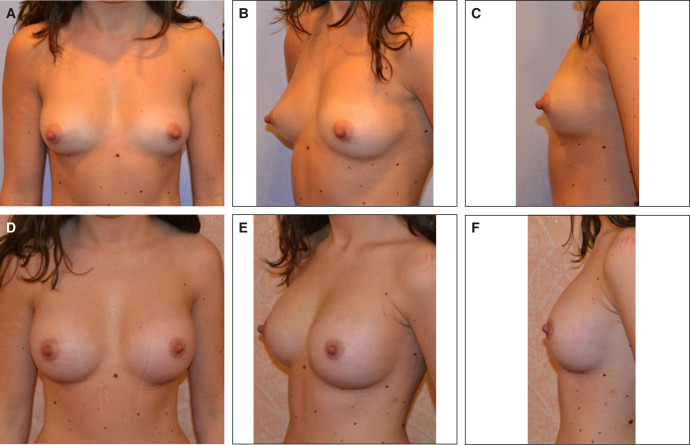
Patient 2 (a 26-year-old female): (A) before operation, frontal view; (B) before operation, oblique view; (C) before operation, lateral view; (D) 12 months after operation, frontal view; (E) 12 months after operation, oblique view; and (F) 12 months after operation, lateral view.

A significant majority of patients, approximately 89%, expressed great satisfaction with the achieved cosmetic result (Figure 6A-F). However, 10% found the outcome acceptable but had higher expectations regarding the shape or size of the breast. Notably, there were no instances of complete dissatisfaction with the results (Table 7).

**Table 7.** Result: Patient Satisfaction Questionnaire

	Score	12 months postoperative
Not satisfied	0	0
	2	0
	4	0
Satisfied	6	21 (10.5%)
Very satisfied	8	127 (63.5%)
	10	52 (26%)

### Third End Points

None of the patients experienced postsurgical complications in the short, medium, or long term (12 months).

## DISCUSSION

Breast augmentation is one of the most common plastic surgery procedures worldwide. However, when conducted under general anesthesia, it carries risks,^24^ such as thromboembolism and potential fatalities,^5^ and requires a lengthy postoperative recovery period.^25^ These factors deter some females from undergoing the procedure, especially those who cannot afford prolonged work absences or are apprehensive about general anesthesia. As a result, the use of tumescence anesthesia in outpatient settings has become more prevalent.^26-28^ We do not use tumescent anesthesia, because this alters the surgical plane, soaks the tissues, and makes the use of the electrocoagulator more difficult. Furthermore, there is a risk of systemic reabsorption of the local anesthesia. Leveraging our extensive experience in peripheral nerve blocks,^29-31^ we have devised a loco-regional anesthesia technique. This method allows patients to remain conscious or only mildly sedated during the operation. Our research findings show that this technique expedites postoperative recovery, enabling hospital discharge within 3 h. Although this technique may have potential risks, such as systemic diffusion of local anesthesia and pneumothorax, we have not detected any complications. This can be justified by the fact that the procedure is performed under ultrasound guidance, and the right plane between the major and the minor pectoralis muscles is clearly identified and injected.

We adapted the breast enhancement method originally described by Tebbetts and Adams.^32-39^ Initially, we made a meticulous incision along the natural mammary fold without any alterations at the base. With time, the implants adjusted and assumed the shape of a new fold. Furthermore, we created an implant pocket smaller than the prosthesis, resembling a vertical tubule. This allowed the prosthesis to adapt to the pocket size for an ideal fit after insertion. Next, we preserved a segment of the lateral pectoralis major muscle to stabilize the prosthesis and prevent lateral displacement. In the closing procedures, we opted not to seal the mammary fold's fascia but merely closed the subcutaneous space and skin. The 1-year outcomes demonstrate that the shape of the breasts and the placement of the prosthesis are excellently preserved.

Endoscopic-assisted transaxillary breast augmentation using a dual-plane implant position is a commonly employed technique.^40-42^ However, it necessitates general anesthesia, involves a steep learning curve, and carries potential complications in the axillary region.^43^ We have modified this technique to use a skin incision at the mammary fold. In our view, this endoscopic approach to preparing the implant pocket lessens surgical trauma.

The pectoralis major muscle is detached from the thoracic fascia and pectoral minor muscle in a nontraumatic manner using monopolar forceps and clear visibility. The release of the sternocostal pectoralis major fibers is meticulously precise. A minimum quantity of fibers is sectioned to prevent muscle animation, while retaining enough fibers to provide medial support. This careful balance ensures the prevention of implant palpability and medial rippling. In our experience, no patient reported any occurrence of rippling or palpability of the implant at the 12-month mark.

Choosing to use smaller implants makes sense, considering that the patients started with an A cup and desired an increase of only 1 or 2 cup sizes. A larger volume could potentially add excessive strain to the muscle and skin, possibly resulting in increased postsurgical pain, extended recovery periods, and a higher risk of complications. To ascertain the risk levels associated with the size of the implants, a thorough scientific study examining these parameters is required.

To assess the procedure's safety and efficacy, we set strict inclusion criteria aiming at minimal bias, particularly considering the patients’ young age and implant size. In fact, we selected females only under the age of 40 based on our experience that in the 18 to 40 age group, the risk of systemic pathologies is statistically lower and patients have a fast recovery. Again, the maximum size of the breast prosthesis was chosen to be 295 cc on the basis of the technical possibility of being able to insert it through a small lower incision of less than 3 cm in the mammary fold through the use of the Keller funnel.

None of the patients required pain medication up to 12 h postsurgery. Mild postoperative pain was reported by 76% of patients at 12 h and 13% at 48 h. Only a quarter of patients characterized the pain as moderate, declining to 2% at 48 h. Notably, no patient needed pain relievers after 72 h postsurgery.

The loco-regional anesthesia likely contributed to pain coverage for up to 12 h postsurgery. This seemed to limit the need for substantial doses of painkillers, presumably due to the minimally invasive technique used to prepare the prosthetic pocket. Additionally, we gained the impression that the small size of the skin scar helped to lessen discomforts such as burning and tension, which are common after surgery when the scar is larger. Also, the psychological benefit of knowing that they had only a small scar, less than 3 cm, might have also helped patients. The avoidance of drainage and compression bandages could also be a contributing factor to this outcome.

Patients recovered quickly postoperation, with all of them discharged from the clinic within 3 h following the procedure. They returned to work within 24 h and began driving after 48 h. All patients resumed their presurgery activities within 7 days of the operation. Within 2 weeks, they returned to sports activities involving the abdomen and lower limbs, and within a month, to activities that also engaged the arms and chest.

Tebbetts and Adams described their procedure as characterized by a lack of pain and quick recovery after surgery, but the technique was performed under general anesthesia; they did not report the time to return to a normal life, including sports, and they reported complications.

In our study, after a 12-month follow-up, no patient reported complications. The bloodless endoscopic atraumatic technique, which does not require the use of drains,^44,45^ may have contributed to the absence of hematoma episodes. The loco-regional anesthesia utilized helped maintain stable blood pressure of the patients during the operation, preventing postoperative fluctuations that could cause blood vessels to open and accumulate serum-blood collections.

In our series, no patient experienced early or late seroma. This outcome could be attributed to both the minimal trauma that resulted during pocket preparation and the adaptation of the periprosthetic pocket to the prosthesis. This fit prevents implant displacements and friction during arm movement, which could otherwise cause serosity. Additionally, the lateral muscle sling enhances implant stability, thereby preventing postoperative dislocations and friction.

None of the patients experienced bottoming out or implant dislocation in the inframammary fold, although we did not suture the fascia. This is a common complication in the dual-plane breast augmentation procedure.^46,47^

There have been no reported instances of postoperative infection following the procedures we have performed, although we have not administered any antibiotics in the periprosthetic pocket.^48-51^ This outcome can be attributed to several factors: first, the utilization of local anesthesia, which averts the immune system depression commonly associated with general anesthesia, thus promoting a swift recovery; second, the minimally invasive and bloodless nature of the endoscopic technique; third, the incorporation of the Keller funnel effectively reduces contamination to a bare minimum;^52,53^ lastly, the deliberate avoidance of sizers may have provided additional positive benefits.^54^

The intervention's final evaluation after 12 months was considered excellent. This comprehensive assessment includes satisfaction with the outcome's aesthetics, including the shape and size of the breasts. However, the experience of the patients with the operation may have also influenced the judgment.

The experience of staying awake during the operation, returning home in just a few hours, having dinner with family the same evening, and resuming work and family duties the next day may all have contributed to the overall satisfaction of the patients. Indeed, 63% of patients would recommend this procedure to their acquaintances.

However, this technique also has several limitations. First of all, it requires an anesthetist who is trained in the pectoral muscle's ultrasound-assisted nerve block; furthermore, it presents potential risks such as systemic diffusion of the local anesthesia and pneumothorax. Again, it requires a surgical learning curve in the use of the endoscope. Finally, it has very limited indications, as described in the inclusion criteria of our study.

## CONCLUSIONS

In our initial study, we showed that endoscopic breast augmentation conducted under localized anesthesia is a safe procedure. It allows for quick recovery postsurgery and swift resumption of everyday activities. The overall complication risk is less than what has been reported, in scientific studies, for the classic dual-plane technique. Moreover, this approach yields excellent patient satisfaction. This technique has a very limited and strict set of indications and also several limitations. Additional prospective and randomized studies will be required to enhance the scientific validity of this technique. Moreover, a larger patient cohort will be essential to stratify the risks associated with varying prosthetic volumes.
